# Leg Motion Classification with Artificial Neural Networks Using Wavelet-Based Features of Gyroscope Signals

**DOI:** 10.3390/s110201721

**Published:** 2011-01-28

**Authors:** Birsel Ayrulu-Erdem, Billur Barshan

**Affiliations:** Department of Electrical and Electronics Engineering, Bilkent University, Bilkent, 06800 Ankara, Turkey; E-Mail: ebirsel@gmail.com

**Keywords:** leg motion classification, inertial sensors, gyroscopes, accelerometers, discrete wavelet transform, wavelet decomposition, feature extraction, pattern recognition, artificial neural networks

## Abstract

We extract the informative features of gyroscope signals using the discrete wavelet transform (DWT) decomposition and provide them as input to multi-layer feed-forward artificial neural networks (ANNs) for leg motion classification. Since the DWT is based on correlating the analyzed signal with a prototype wavelet function, selection of the wavelet type can influence the performance of wavelet-based applications significantly. We also investigate the effect of selecting different wavelet families on classification accuracy and ANN complexity and provide a comparison between them. The maximum classification accuracy of 97.7% is achieved with the Daubechies wavelet of order 16 and the reverse bi-orthogonal (RBO) wavelet of order 3.1, both with similar ANN complexity. However, the RBO 3.1 wavelet is preferable because of its lower computational complexity in the DWT decomposition and reconstruction.

## 1. Introduction

Sensor networks, particularly wireless sensor networks, have received considerable attention since the recent significant advances in sensor technologies, micro-electro-mechanical systems (MEMS), wireless communications, and distributed signal processing technology [1,2]. A wireless sensor network is a collection of arbitrarily distributed tiny, low-cost, battery-powered sensor nodes that can collect data from the surrounding area, carry out simple computations, and communicate with each other or a base station. These networks are especially attractive in risk-associated applications such as habitat monitoring and environmental observation, disaster detection and prediction, health monitoring, and military applications. Wireless sensor networks have some advantages over traditional sensing configurations in terms of reliability, accuracy, and cost effectiveness. Easy and dense deployment of these networks not only extends the spatial coverage and achieves higher resolution, but also increases their fault-tolerance and robustness properties.

When sensors are deployed in or on the human body, these networks are called wireless body area networks (WBANs), which can be considered a subdivision of the wireless sensor networks applied in the health-care domain [3–5]. An extensive set of physiological sensors consisting of wearable and implantable units can be incorporated into WBANs, such as electrocardiograms (ECG), electromyography (EMG), and electroencephalography (EEG) sensors to monitor heart, muscle, and brain electrical activity, respectively. Data from blood pressure sensors, respiration sensors, tilt sensors for monitoring the trunk position, and miniature inertial motion sensors such as accelerometers and gyroscopes can be used in WBANs to predict human state and activity. Wireless body area networks offer flexibility and mobility to patients by allowing an ongoing patient monitoring system in hospitals and residential and working environments, crucial for diagnosis and prevention purposes and optimal maintenance of some chronic conditions. In patient monitoring systems, classifying human activity is necessary to record under which conditions medical data are acquired. Human activity recognition is also important in applications such as surveillance and security of the elderly who live alone, with an aim to improve their quality of life, independence, safety, and mobility, as well as the speed of health care services provided to them. Comprehensive surveys on human activity recognition can be found in our earlier works [6,7].

Multi-axial accelerometers have been used for human activity recognition in numerous existing works [8–11]. These sensors have been proven to function well in identifying human activity containing intense actions such as walking, jogging, jumping, *etc*. For sedentary activities such as sitting, standing, and lying down or for activities in which orientation is important, traditional accelerometer-based solutions do not work very well. To eliminate the deficiencies of accelerometer-based solutions, these sensors are used in conjunction with other sensing modalities such as proximity sensors and gyroscopes [12].

In any classification or recognition problem, feature extraction is an important process to identify those features with relatively small intra-class and large inter-class variations. Since such features are more discriminative, they result in more accurate classification. Furthermore, a smaller number of features reduces the computational burden of the classification process. In feature extraction, the processing of signals is necessary for the awareness of different contexts of these signals in different domains. Wavelet transform is a decomposition technique with considerable time localization advantages over the frequently used Fourier-based transformation techniques that extract the frequency content of signals. The wavelet transform technique is also advantageous over traditional Fourier methods in analyzing physical behaviors where the corresponding signals may contain discontinuities and sharp spikes [13].

In this paper, we use small, low-cost gyroscopes positioned on the human leg to classify leg motions. The motivation behind classifying leg motions is the potential application in physiotherapy and home-based rehabilitation. For example, a patient with paralysis may be given certain exercises to do regularly, and inertial sensors can be used remotely to assess which exercise the patient is performing and whether he is doing it properly. Several different feature sets based on the discrete wavelet transform (DWT) decomposition and their combinations are considered for effective feature extraction. The set that gives the highest classification accuracy and minimum artificial neural network (ANN) complexity is identified and used. Raw gyroscope signals, initially comprised of a large number of samples (1, 600 × 2), are represented by a moderate number of highly informative features (12). This enables us to use ANNs as motion classifiers with high accuracy and relatively low network complexity. Since the DWT correlates the analyzed signal with a prototype wavelet function, selection of the wavelet function is a critical process that influences the performance of any wavelet-based application. Thus, in this work, we also investigate the effect of different types of wavelet functions on classification performance and provide a comparison between them.

This paper is organized as follows: In Section 2, we introduce the motions classified in this study and describe the experimental methodology. A brief review of the DWT and its use in signal decomposition is provided in Section 3. The ANNs used in this study are described in Section 4. Different features extracted based on the DWT decomposition of gyroscope signals are described and their performance on leg motion classification with ANNs is presented in Section 5. In Section 6, the effect on classification accuracy of choosing different wavelet families for the DWT is summarized. Results are presented and discussed in Section 7. Concluding remarks are made and directions for future research are suggested in the last section.

## 2. Classified Leg Motions and Experimental Methodology

**Figure 1.**
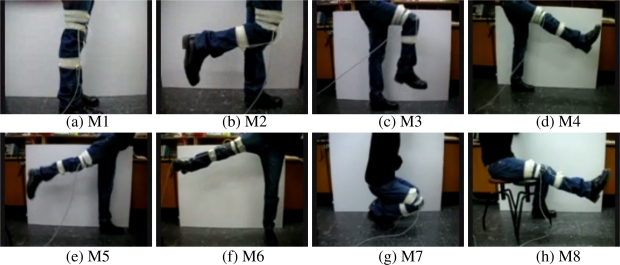
Eight different leg motions.

Eight different sample leg motions are classified using two single-axis gyroscopes that are placed on the subject’s right leg. Photos taken while performing the motions are shown in Figure 1. Throughout the motions listed below, the subject’s left foot stays on the ground. The motions are:

M1: standing without moving the legs (Figure 1(a)),
M2: moving only the lower part of right leg to the back (Figure 1(b)),
M3: moving both the lower and the upper part of the right leg to the front while bending the knee (Figure 1(c)),
M4: moving the right leg forward without bending the knee (Figure 1(d)),
M5: moving the right leg backward without bending the knee (Figure 1(e)),
M6: opening the right leg to the right side of the body without bending the knee (Figure 1(f)),
M7: squatting, moving both the upper and the lower leg (Figure 1(g)),
M8: moving only the lower part of the right leg upward while sitting on a stool (Figure 1(h)).

**Figure 2.**
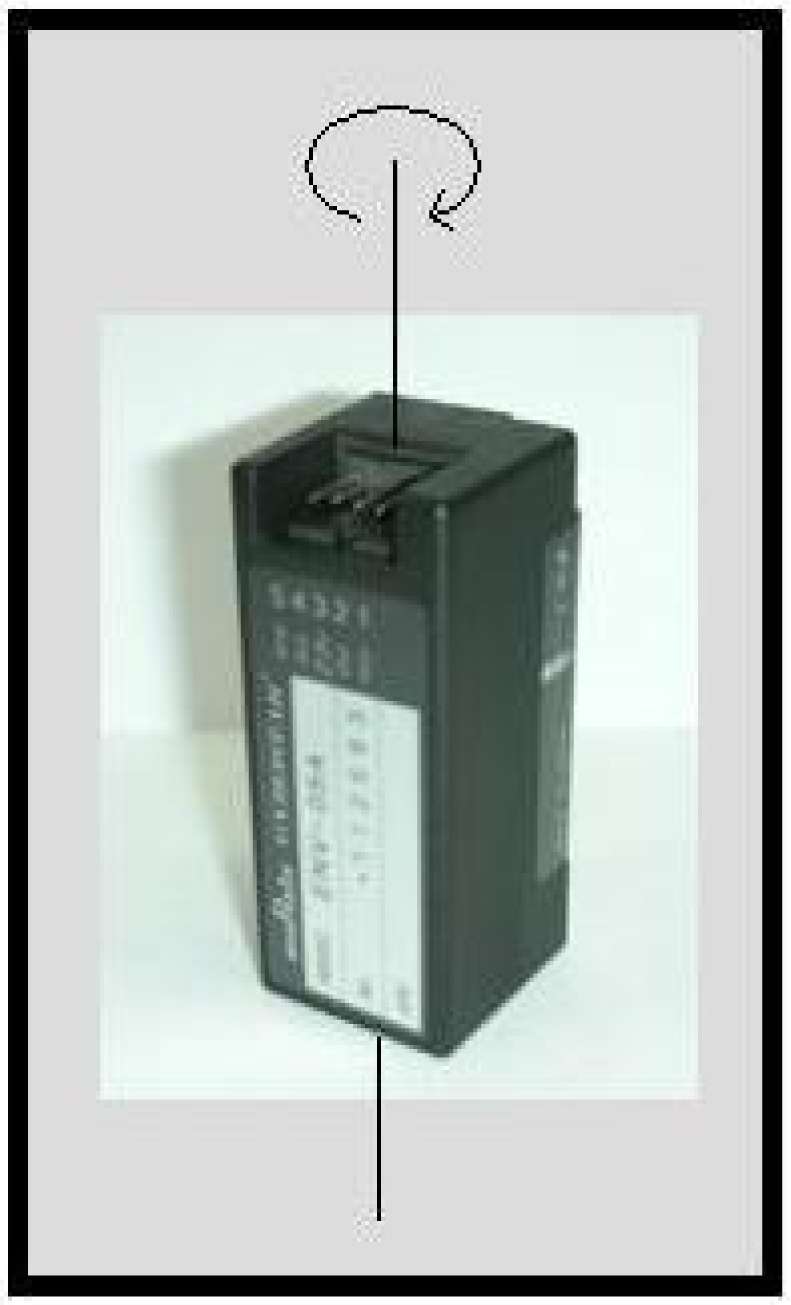
Murata Gyrostar ENV-05A.

The two gyroscopes used are Gyrostar ENV-05A piezoelectric vibratory gyroscopes manufactured by Murata (Figure 2). The Gyrostar is a small, relatively inexpensive piezoelectric gyro originally developed for the automobile market and active suspension systems [14]. The main application of this device has been in helping car navigation systems keep track of turns when, for short durations, the vehicle is out of contact with reference points derived from additional sensors. The Gyrostar consists of a triangular prism made of a substance called “Elinvar”, on each vertical face of which a piezoelectric transducer is placed. Excitation of one transducer perpendicular to its face at about 8 kHz causes vibrations to be picked up by the other two transducers. If the sensor remains still or moves in a straight line the signals produced by the pick-up transducers are exactly equal. If the prism is rotated around its principal axis, Coriolis forces proportional to the rate of rotation are created.

This device operates with a DC supply voltage between eight and 13.5 V and converts angular velocity information to an analog DC voltage at its output [15]. The output voltage is proportional to the angular velocity of the device around its principal axis and varies between 0.5 and 4.5 V. The maximum rate that can be measured with the Gyrostar is *±*90°/s. An angular velocity of zero (no motion) corresponds to a voltage output of 2.5 V. At the maximum angular velocities of +90°/s and −90°/s, the output voltages become 4.5 V and 0.5 V, respectively. If the angular velocity is larger than the maximum value (*±*90°/s), saturation occurs at the corresponding voltage level (0.5 or 4.5 V) so that the rate and the orientation information become erroneous and need to be reset.

**Figure 3.**
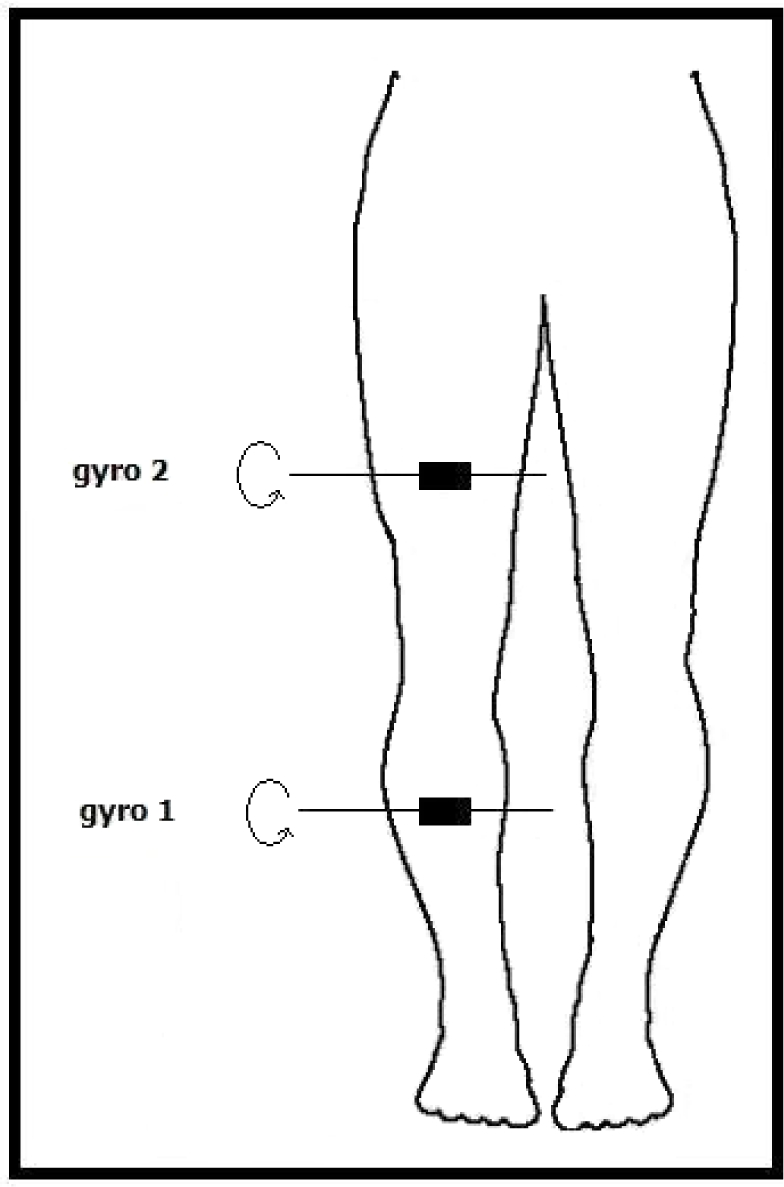
Position of the two gyroscopes on the human leg (body figure adopted from http://www.answers.com/body breadths).

Because these devices are sensitive to rotations around a single axis, positioning these sensors must take their sensitivity axes into account. For our purposes, one of the gyroscopes is placed 17 cm above and the other one 15 cm below the right knee of the subject, as illustrated in Figure 3. The sensors’ sensitivity axes are placed parallel to the ground and to the front of the body. In this way, the highest number of different motions can be detected.

**Figure 4.**
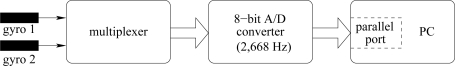
Block diagram of the experimental setup.

The block diagram of the experimental setup is given in Figure 4. It comprises two piezoelectric gyroscopes for sensing the leg motions, a multiplexer to multiplex the signals of the two gyros, an eight-bit analog-to-digital (A/D) converter with a sampling frequency of 2,668 Hz, and a PC. Data acquired by the A/D converter is recorded on the PC through the parallel port of the computer with a simple interface program written in Turbo C++. After acquiring and storing this data, the signals are downsampled by 20 to obtain 133.4 Hz digital signals. Sensor signal processing is done using MATLAB.

In a laboratory environment, a male subject performs the above eight motions. The duration of each motion is about five to seven seconds and 10–14 repetitions of the same motion are made over a period of 72 s. The motion is repeated for seven more 72-second intervals. The subject then performs the next motion for the total of eight 72-second intervals. In the end, the total signal duration per leg motion is approximately 576 (= 8 *×* 72) seconds.

**Figure 5.**
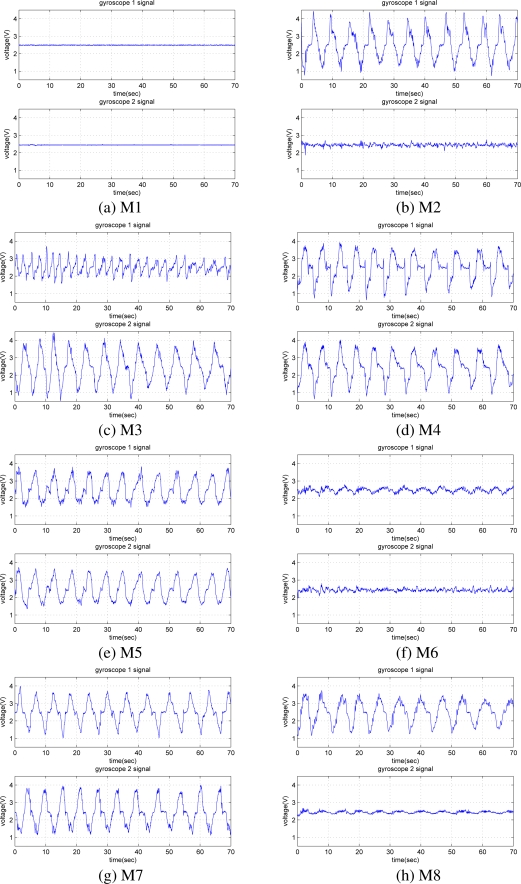
Sample signal recordings of the two gyroscopes for each type of motion. The repetitive patterns in the subfigures are caused by repeating the motion 10–14 times.

Each 72-second signal is divided into six 12-second segments. Hence, while acquiring signals for each motion, a total of 48 (= 6 segments *×* 8 repetitions) 12-second segments are recorded from each gyroscope. Each signal segment consists of 1,600 samples. As there are two gyroscopes, 96 (= 48 *×* 2) signal segments are available for each motion. Thus, a total of 768 (= 96 segments *×* 8 motions) signal segments are available. Sample gyroscope signals for eight different leg motions are given in Figure 5, where the quasi-periodic nature of the signals can be observed.

## 3. Basics of the Wavelet Transform

The wavelet transform is commonly used in signal processing applications such as compression, encoding, denoising, feature extraction, decomposing, and reconstructing signals [16]. The Fourier transform retrieves only the global frequency content of a signal, whereas the wavelet transform has the ability to perform local analysis and has several advantages over traditional Fourier methods in analyzing the signals that are highly non-stationary, noisy, and aperiodic.

Two basic wavelet transforms are the continuous and the discrete wavelet transforms. Any decomposition of a signal into a set of basis functions called *wavelets* involves a pair of waveforms that represent the high frequencies corresponding to the details of a signal, named the *wavelet function*, and the low frequencies or the smooth parts of a signal, called the *scaling function*. A wavelet is a short, oscillating function including both the analysis function and the window. Time information is obtained by shifting the wavelet over the signal and correlating the two. The frequencies are changed by contracting and dilating the wavelet function.

**Figure 6.**
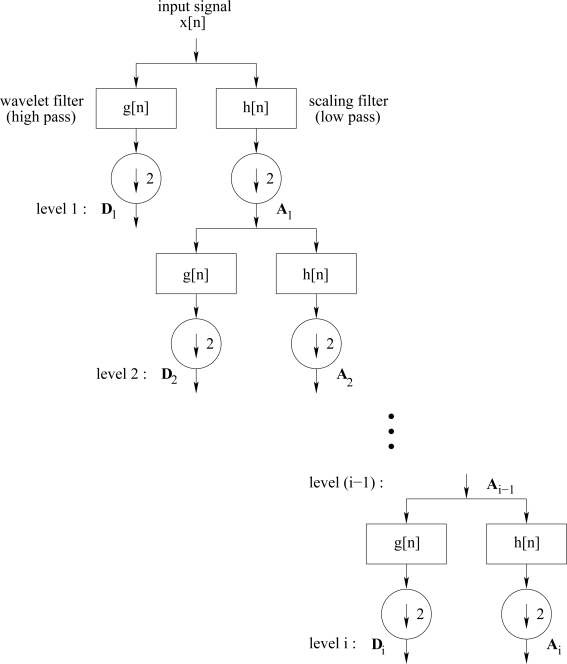
Decomposition of a signal *x*[*n*] with DWT at level *i*.

In the case of the DWT, any signal can be decomposed into a set of discrete wavelet coefficients using wavelets. Generally, the DWT uses filter banks for the analysis and synthesis of a signal. The filter banks contain wavelet and scaling filters to extract the frequency content of the signal in various sub-bands [17]. More specifically, the DWT initially decomposes a discrete signal into approximation and detail coefficients by filtering it through scaling and wavelet filters, respectively, and then downsampling the resulting sub-signals by two. The approximation coefficients are subsequently divided into new approximation and detail coefficients using the same process. This process is iteratively carried out to produce a set of global approximation coefficient vectors **A***_i_* and detail coefficient vectors **D**_1_, **D**_2_,…,**D***_i_* at the *i*th level, as illustrated in Figure 6. If the decomposed signal has *N* samples, at the *i*th level, the row vector **A***_i_* has $\frac{N}{2^{i}}$ elements and the row vectors **D***_j_* have $\frac{N}{2^{j}}$ elements, where *j* = 1,…,*i*. The properties and a performance comparison of the wavelet families commonly used in DWT is provided in Section 6 for our problem, with some historical background.

## 4. Multi-layer Feed-Forward Artificial Neural Networks

In this section, we review the basics of ANNs that will be employed for leg motion classification using the wavelet decomposition of gyroscope signals at their input. Artificial neural networks have been widely and efficiently used for classification problems in applications such as target detection and tracking [18], speech processing [19], system identification [20], control theory [21], medical applications [22], and character recognition [23]. In these and similar applications, multi-layer feed-forward ANNs have been preferred more often than any other type of ANN because of their capability of learning nonlinear mappings, being non-parametric, and making weaker assumptions on the shape of the underlying distribution of the input data than other statistical classifiers [24–26]. The performance of ANNs in any application is affected by the choice of the network type, parameters of the network structure, input signal given to the network, type of training method and algorithm, as well as parameter initialization. Network complexity is very much affected by the size and the type of the input signal.

The typical multi-layer feed-forward ANN is composed of an input layer, one or more hidden layers, and a single output layer, each comprised of a number of units called neurons. Two well-known methods for determining the number of hidden-layer neurons in ANNs are *pruning* and *enlarging* [25]. Pruning begins with a relatively large number of hidden-layer neurons and eliminates unused neurons according to some criterion. Enlarging begins with a relatively small number of hidden-layer neurons and gradually increases the number until the maximum possible learning rate is achieved with the training data.

Artificial neural networks have three distinctive characteristics: The model of each neuron includes a smooth nonlinearity, the network contains one or more hidden layers to extract progressively more meaningful features, and the network exhibits a high degree of connectivity. Due to the presence of the distributed form of nonlinearity and the high degree of connectivity, theoretical analysis of multi-layer perceptrons is difficult. These networks are trained to compute the boundaries of decision regions in the form of connection weights and biases by using training algorithms. The operation of ANNs is characterized by two phases, training and testing. Training can be achieved using either *supervised* or *unsupervised* training methods. In supervised training, a set of *training patterns* is provided to the network as input and propagated forward to determine the resulting signal at the output. The corresponding desired outputs are also readily provided to the ANN. The weights and biases, which are the parameters of the network, are adjusted to get the desired outputs from the inputs by employing a supervised training algorithm. In unsupervised training, only the inputs are provided to the ANN and the training algorithm adjusts the parameters of the network by grouping similar input patterns to the same output nodes. The number of classes is determined by the number output nodes. In the test phase, test data similar to the training patterns are used as input, and the ANN predicts the response to the test data based on the learned response. In this work, supervised training and three-layer feed-forward ANNs trained with the Levenberg-Marquardt algorithm [27,28] are employed. This algorithm is an effective second-order approach proposed to speed up the widely used classical error back-propagation algorithm and its heuristically modified versions such as the error back-propagation algorithm with momentum constant, variable learning rate, and stochastic learning in ANN training.

In this study, the number of neurons in the input layer of the ANN is selected to be equal to the number of elements used in each extracted feature. The number of hidden-layer neurons is found by enlarging, briefly described above. The number of output neurons is equal to the number of different leg motions, which is eight in our case. After considering different activation functions in the hidden and output layers, better classification performance is obtained when a logarithmic sigmoid function is used as the activation function in all hidden-layer neurons and when linear neurons are engaged at the output layer. The desired outputs are coded such that the *i*th output neuron is set to one if the input data given to the ANN belongs to motion *i*; the rest of the output nodes are set to zero, where *i* = 1,…,8. The decision for testing the data at the output layer is made based on the maximum selection rule; if the *i*th output neuron has the maximum value, this indicates that the input data given to the ANN belongs to motion *i, i* = 1,…,8. The MATLAB Neural Network Toolbox is employed for the implementation and training of these networks [29]. One third of the 768 patterns are randomly selected to be used as training data and the rest are assigned as test data.

## 5. Leg Motion Classification Based on the DWT and ANNs

**Figure 7.**
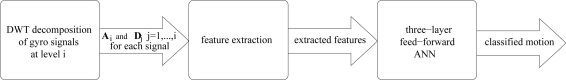
The block diagram of the processing stages using DWT decomposition and ANNs.

Feature extraction involves identifying the most informative features in a given pattern [30]. Features with smaller variations between similar patterns (intra-class variation) and larger variations between the different types of patterns (inter-class variation) are favorable. Selecting the most discriminative features is crucial; otherwise, patterns will not be recognized efficiently and the misclassification rate will be higher. In this work, different features of the gyroscope signals are extracted using the DWT and used prior to ANNs in the leg motion classification process. A block diagram of the processing stages is given in Figure 7.

The Daubechies wavelet of order four is chosen as the mother wavelet in the DWT computations used for wavelet-based feature extraction. This wavelet is widely used in signal processing applications because it is continuous with a continuous first-order derivative and is relatively simple compared to higher-order Daubechies wavelets. Levels one through eight of the DWT decomposition are investigated.

The total energy *E_T_* at level *i* of the DWT decomposition is given by:

(1) $$E_{T}={\text{A}}_{i}{\text{A}}_{i}^{T}+\sum_{j=1}^{i}{\text{D}}_{j}{\text{D}}_{j}^{T}$$

One feature that may be significant is the ratio of the energy allocated to each type of coefficient to the total energy of the DWT coefficients. From this point on, we will refer to this ratio as the energy distribution ratio (EDR) and distinguish between EDR**_A_** and EDR_**D**_*j*__ as the EDRs of the approximation and detail coefficients, respectively:

(2) $${\text{EDR}}_{\text{A}}=\frac{\text{energy}\;\text{of}\;\text{the}\;\text{approximation}\;\text{coefficient}\;\text{vector}\;\text{at}\;\text{level}\;i}{\text{total}\;\text{energy}\;\text{of}\;\text{the}\;\text{coefficient}\;\text{vectors}\;\text{at}\;\text{level}\;i}=\frac{{\text{A}}_{i}{\text{A}}_{i}^{T}}{E_{T}}$$

(3) $${\text{EDR}}_{{\text{D}}_{j}}=\frac{\text{energy}\;\text{of}\;\text{the}\;\text{detail}\;\text{coefficient}\;\text{vector}\;j\;\text{at}\;\text{level}\;i}{\text{total}\;\text{energy}\;\text{of}\;\text{the}\;\text{coefficient}\;\text{vectors}\;\text{at}\;\text{level}\;i}=\frac{{\text{D}}_{j}{\text{D}}_{j}^{T}}{E_{T}}\;\;\;j=1,\ldots ,i$$

The features of the two gyroscope signals that we have considered and evaluated are given below. For each feature set, the range of motion classification accuracies achieved with the test data set and the levels at which the minimum and maximum values are obtained are given in parentheses, respectively.

- normalized approximation (**A**) coefficients (41.4%–68.2%, levels 4 and 7)
- normalized approximation (**A**) and detail (**D**) coefficients (16.7%–58.7%, levels 4 and 8)
- EDR_**D**_*j*__’s (18.2%–85.2%, levels 1 and 7)
- EDR**_A_**’s and EDR_**D**_*j*__’s (21.2%–86.4%, levels 1 and 6)
- EDR**_A_**’s and the minimum, maximum, mean, and variance of the EDR_**D**_*j*__’s (19.7%–84.1%, levels 1 and 6)
- the minimum, maximum, mean, and variance of the EDR_**D**_*j*__’s (23.1%–79.2%, levels 1 and 6)
- EDR**_A_**’s and the mean and the variance of the EDR_**D**_*j*__’s (21.5%–79.2%, levels 1 and 5)
- the mean and the variance of the EDR_**D**_*j*__’s (23.9%–78.0%, levels 1 and 5)
- the normalized means and variances of the DWT decomposition coefficients (73.1%–87.5%, levels 8 and 3)
- the normalized means of the DWT decomposition coefficients (16.7%–32.0%, levels 3 and 1)
- the normalized variances of the DWT decomposition coefficients (78.5%–95.1%, levels 1 and 5)
- the normalized variances and the EDRs of the DWT decomposition coefficients (82.4%, 95.1%, levels 7 and 5&6)

The normalized means and variances of the DWT decomposition coefficients are calculated over the elements of each DWT coefficient vector at the *i*th level (**A***_i_*, **D**_1_, **D**_2_,…,**D***_i_*). Therefore, at the *i*th level, *i* + 1 mean and *i* + 1 variance values are calculated for each gyro signal segment, totaling 2(*i* + 1) features. Since the signals originate from two gyros, the number of neurons at the input layer of the ANN is 4(*i* + 1). The results for this feature set are presented in Table 1. In the table, the ANN complexity is given in *n*_1_:*n*_2_:*n*_3_ format, where the three numbers represent the number of neurons in the input, hidden, and output layers of the network, respectively. Referring to this table, 100% classification accuracy with the training set is achieved for all levels and the maximum classification rate of the test set is 87.5%. This value is obtained at level three with a network size of 16:16:8. Notably, with this feature set, the classification accuracies for levels one to four are all above 82% with the test data. After level four, although the number of elements used in the feature set increases, the classification accuracy does not improve; the minimum classification accuracy of 73.1% is acquired at level eight with a network complexity of 36:10:8.

The highest classification accuracies are obtained with the feature set consisting of the normalized variances of the DWT decomposition coefficients. The normalized variances of the DWT decomposition coefficients are observed to be much more informative than their normalized means. The detailed results obtained by using this feature set are given in Table 2. The maximum classification accuracy (95.1%), by employing only the normalized variances of the DWT decomposition coefficients, is achieved at the fifth decomposition level and is even higher than that obtained by the joint use of normalized means and variances of these coefficients (87.5%). This is also the maximum classification accuracy among all the feature sets considered. With this feature set, the minimum correct classification rate increases to 78.5% and for most of the levels a success rate of around 90% is achieved with the test data.

Among all the feature sets considered in this study, the two that result in the highest classification rates are the normalized variances and the EDRs of the DWT decomposition coefficients. Thus, we also consider the joint use of these two feature sets to explore whether a classification accuracy above 95.1% can be achieved. These results are given in Table 3. In this case, although considerable increase in classification accuracy is observed for the majority of the levels, the highest classification accuracy still remains 95.1%, and at the cost of doubling the number of input-layer neurons and adding two more hidden-layer neurons in each ANN (compare the last columns of Tables 2 and 3). For this reason, we carry out the rest of our research with the feature set whose elements are the normalized variances of the DWT decomposition coefficients of the two gyro signals at the fifth level.

**Figure 8.**
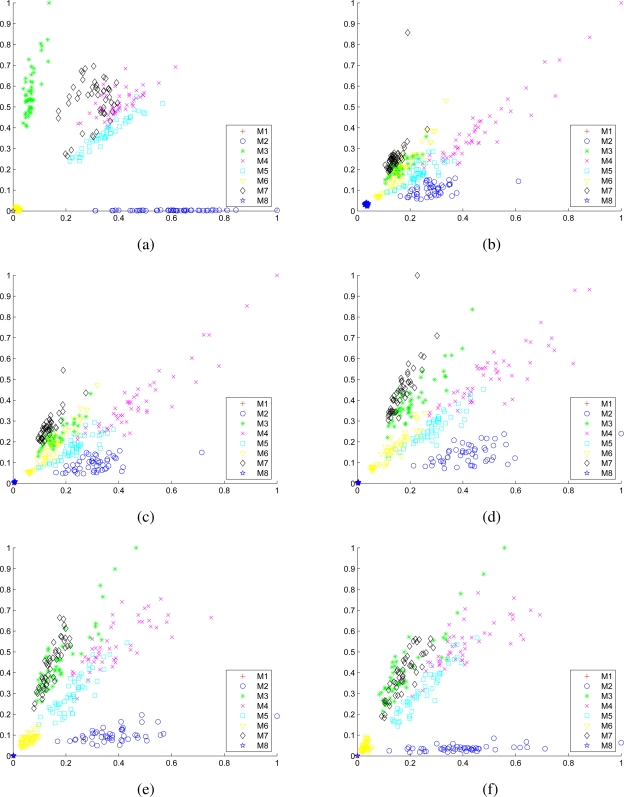
Scatter plots of the normalized variances of (a) **A**_5_, (b) **D**_1_, (c) **D**_2_, (d) **D**_3_, (e) **D**_4_, and (f) **D**_5_ over all motion types. Features extracted from the first and the second gyro signals are plotted along the horizontal and the vertical axes of the plots, respectively.

Scatter plots of the 12 members of the selected feature set consisting of the normalized variances of the DWT decomposition coefficients of the two gyro signals for all eight motions are provided in Figure 8. In each scatter plot, the horizontal and vertical axes correspond to the normalized variance of one of the DWT decomposition coefficients of the signal acquired with the first and the second gyroscopes, respectively.

## 6. Wavelet Selection

Since the mother wavelet produces all the wavelet functions that determine the characteristics of the resulting wavelet transform, its choice is critical and directly related to the efficient use of this transform in a given application. There is no systematic way to select the mother wavelet and the choice depends on the application at hand. The wavelets can constitute an orthogonal or bi-orthogonal (BO) basis, or a simple frame. In selecting the wavelet, the most important and commonly considered properties are the regularity, number of vanishing moments, symmetry, and compactness. The regularity or the number of continuous derivatives indicates how smooth the wavelet is. The localization capability of the wavelet in the frequency domain is directly related to the regularity such that the larger the regularity, the sharper the Fourier transform of the wavelet in the frequency domain. The number of vanishing moments is related to the number of oscillations of the wavelet (*i.e.*, localization in time). More importantly, if a wavelet has *M* vanishing moments, the wavelet transform can be interpreted as a multi-scale differential operator of order *M*. This produces the relation between the differentiability of a signal and its wavelet transform decay at fine scales, which is a useful property for compression applications. The symmetry means that the filters have a linear phase, which is a key property to provide perfect reconstruction. The size of the support of the wavelet (compactness) is also important such that the wider this support, the higher the computational power required for the wavelet transform. The maximum number of vanishing moments is proportional to the size of the support of the wavelet such that if a wavelet is *M* times differentiable, it has at least *M* vanishing moments. Thus, a trade-off between computational power and analysis accuracy as well as between time and frequency resolution exists in a given problem.

Haar [31] described the first and the simplest wavelet basis in 1910. This wavelet is discontinuous and resembles a step function. In 1981, the transformation method of decomposing a signal into wavelet coefficients and then reconstructing the original signal based on these coefficients was derived by Grossman and Morlet [32]. After this date, different researchers have developed a wide variety of wavelets. In 1985, Meyer constructed the first non-trivial wavelet, which is continuously differentiable but does not have a compact support. Meyer’s scaling and wavelet functions are both defined in the frequency domain. Although these functions are symmetric, a fast algorithm is not available for their wavelet transform [33]. Mallat and Meyer developed a multi-resolution analysis using wavelets in 1986 [34]. Daubechies wavelets, proposed by Ingrid Daubechies around 1989, represent the foundations of wavelet signal processing; they are the most popular [35]. The Haar wavelet has the advantage of being simple to compute and easy to understand. Daubechies wavelets have a slightly higher computational complexity and are conceptually more complex, however, Daubechies wavelets can extract the high-frequency content of a signal better than Haar wavelets. The order of the Daubechies wavelets denotes the number of vanishing moments, or the number of zero moments of the wavelet function.

The most commonly used wavelets in signal processing applications are Haar, Daubechies, Coiflet, Symlet, bi-orthogonal, reverse bi-orthogonal (RBO), Meyer, Morlet, and Mexican hat wavelets. The fundamental properties of these basic wavelet families are summarized in Table 4. The first four are compactly supported orthogonal wavelets. Coiflets and Symlets originate from the Daubechies wavelet [36]. Coiflets have the highest number of vanishing moments for both the wavelet and the scaling function for a given support width. Coiflets are more symmetrical and have more vanishing moments than Daubechies wavelets. Symlets, proposed by Daubechies as modifications to her own wavelet, are the most symmetrical of all. The associated scaling filters are near linear-phase filters. The properties of Symlets are nearly the same as those of Daubechies wavelets. Bi-orthogonal wavelets exhibit the property of symmetry, resulting in linear-phase filters, needed for perfect signal reconstruction. Bi-orthogonal wavelets use two different wavelets; one for decomposition and the other for reconstruction instead of a single one for both. With these two wavelets, interesting properties can be derived. Reverse BO wavelets use the synthesis wavelets of BOs for the analysis part and the analysis wavelets of BOs for the synthesis part of the wavelet transform. Morlet and Mexican hat wavelets only have wavelet functions; the corresponding scaling functions do not exist [37]. These two wavelets can only be used in the continuous wavelet transform.

As stated above, one of the difficulties of using wavelet transform pre-processing is that there is no universal way of choosing the most suitable wavelet for a particular application. In most cases, the best wavelet type can only be found experimentally. In the previous section, we chose the Daubechies wavelet of order four as our mother wavelet due to its wide usage in signal processing applications, continuity of the wavelet function and its first-order derivative, and relative simplicity compared to higher-order Daubechies wavelets. In this section, other types of wavelets besides the Daubechies wavelet of order four are considered and tested, with the feature set constructed by the normalized variances of the DWT decomposition coefficients of the two gyro signals at the fifth level. For the first five wavelet families listed below, the average value and the range of motion classification accuracies achieved with the test set and the orders at which the minimum and maximum values are achieved are given in parentheses, respectively. There is a single result for the last two families since these wavelets do not have different orders.

- Daubechies wavelets of orders two to 32 (average: 89.5%, 76.6%–97.7%, orders 28 and 16)
- Symlets of orders two to eight (average: 90.1%, 81.8%–94.3%, orders 3 and 6)
- Coiflets of orders one to five (average: 93.4%, 87.5%–97.0%, orders 4 and 2)
- BO wavelets of orders 1.1, 1.3, 1.5, 2.2, 2.4, 2.6, 2.8, 3.1, 3.3, 3.5, 3.7, 3.9, 4.4, 5.5, and 6.8 (average: 91.9%, 85.6%–96.6%, BO 3.7 and BO 2.2)
- RBO wavelets of orders 1.1, 1.3, 1.5, 2.2, 2.4, 2.6, 2.8, 3.1, 3.3, 3.5, 3.7, 3.9, 4.4, 5.5, and 6.8 (average: 93.3%, 84.1%–97.7%, RBO 3.3 and RBO 3.1)
- Haar wavelet (91.8%)
- the discrete approximation to the Meyer wavelet (discrete Meyer wavelet) (89.8%)

Among the wavelet families considered in this research, the Haar wavelet is the simplest in terms of the filter length and the processing time, and the discrete Meyer wavelet is the most complex. The complexity of the resulting ANNs is comparable and 12:8:8 in most cases except a few instances where it changes to 12:10:8.

The wavelet families listed above have all resulted in 100% correct classification accuracy using the training data set. Although the average classification accuracy obtained with the wavelet families involved in our research can be considered comparable for the test data set, Coiflets and RBO wavelets result in slightly better classification accuracies on the average, with 93.4% and 93.3%. This is followed by, in descending order, BO wavelets, Haar wavelet, Symlets, discrete Meyer, and Daubechies wavelets. When we consider wavelets of different orders individually rather than in the average sense for each family, Daubechies wavelets of orders 16 and 10, Coiflets of order two, BO 2.2, RBO 3.1, 2.2, 3.5, and 1.3 result in accuracies above 96%. The maximum classification rate achieved in our research is 97.7%, with the Daubechies wavelet of order 16 as well as RBO 3.1, with a similar network complexity of 12:10:8. Although these two wavelets result in the same highest classification accuracy, RBO 3.1 has support widths of three and seven for the decomposition and the reconstruction parts of the DWT, respectively. With the Daubechies wavelet of order 16, this number is 31 for both the decomposition and the reconstruction parts of the DWT. As mentioned at the beginning of this section, the higher the support width, the higher the computational power required for the wavelet transform. For this reason, RBO 3.1 would be a better choice than the Daubechies wavelet of order 16 when the classification accuracy and the computational complexity criteria are considered together.

## 7. Results and Discussion

The effect of random initialization of the ANN parameters are analyzed by generating 10 different ANNs with the same training data set using the RBO 3.1 wavelet. The statistics over these 10 iterations are summarized as a confusion matrix in Table 5(a). In this table, the entry in the *i*th row and the *j*th column corresponds to the mean classification accuracy of actual motion *i* classified as motion *j*, calculated over 10 iterations where *i, j* = 1,…,8. The corresponding standard deviations, calculated in the same manner, are also provided. On the average, a 97.6% classification accuracy with a standard deviation of 1.2% is obtained over all classified motions.

We have also investigated the effect of randomly chosen training and test data sets on classification accuracy. For this purpose, 10 different training and test data sets are randomly constructed. For each training set, a new ANN is generated and tested with the corresponding test data set. The resulting statistics over these 10 iterations are given in terms of the mean and one standard deviation as a confusion matrix in Table 5(b).

Inspecting the confusion matrices in Table 5, we can observe that motion 1 is never confused with any other motion since it is the standing activity. Motion 3 is also classified with high accuracy. Motions 4 and 5 and motions 2 and 8 are the motions mostly confused with each other. Motions 4 and 5 are similar in that both the lower and upper parts of the leg are moving without bending the knee forward and backward, respectively. The confusion of motions 2 and 8 is also caused by their similarity because only the lower part of the leg is moving backward and forward in these motions, respectively.

**Figure 9.**
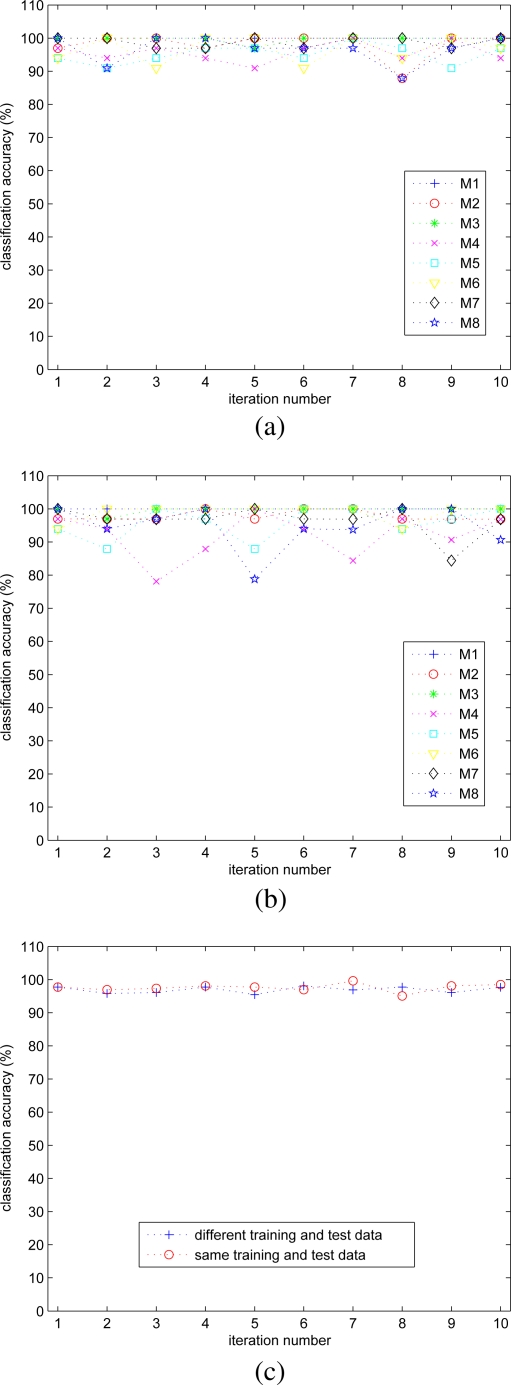
Classification accuracy for each motion type versus the iteration number when (a) the same and (b) different training and test data sets are used in each iteration. (c) The average classification accuracy over all motion types.

In Figure 9(a,b), the classification accuracy for each motion type versus the iteration number is shown when the same and different training/test data sets are used, respectively. For part (a), the minimum, maximum, and mean values of the classification accuracy over all motion types and all iterations are 87.9%, 100%, and 97.6%, respectively. For part (b), the corresponding numbers are 78.1%, 100%, and 96.9%. The standard deviation of the classification accuracy is about 1.0%. In part (c) of the figure, the classification accuracies in parts (a) and (b) are averaged over all motion types and plotted. The results indicate that the effect of random initialization and random selection of training and test data sets on classification accuracy is not very significant.

Pre-processing and classification times are calculated with MATLAB version 7.0.1 on an Intel Centrino + Core @1.6 GHz, 1.024 GB RAM laptop computer running the Microsoft Windows XP Professional operating system. The time it takes to divide the raw gyroscope signals into 12-second segments is 25.6 s. Feature extraction takes approximately 5.4 s and does not change much with the selection of different feature sets. The time it takes to train a network of complexity 12:10:8 with the RBO 3.1 wavelet that results in the highest accuracy is 95.5 s, using the selected features of one third of the signal segments. The testing stage with the features of the remaining two thirds of the signal segments takes about 0.19 s. The training time of the ANN increases with the number of input-layer neurons and the complexity of the network, and can be as high as several hours.

In [6], we use the same data set and initially extract 101 features. However, since not all features are equally useful in discriminating the motions, we reduce the number of features to between 6 and 14 in several different ways: principal component analysis, sequential forward feature selection, and inspection. In that work, six different classifiers are compared, one of them being ANNs trained with the back-propagation algorithm. The maximum classification accuracy achieved with ANNs was 90.2% (see Table 3 in [6]). In the work reported here, we improve the performance of ANNs by pre-processing the inputs using DWT decomposition and providing a more informative and efficient feature set to the network.

An important issue in motion recognition and classification studies is the inter-subject variability caused by the way different individuals perform the same motions. Each person performs a particular motion or activity differently due to differences in body size, gender, style, and timing. In this study, we have demonstrated the proposed methodology using motion data recorded from a single subject. In our different but closely related studies [7,38], we have investigated inter-subject variability where daily and sports activities are performed by eight different subjects in a more naturalistic manner. The subjects were not told exactly how to perform the activities and each subject performed them in their own style and speed. All possible subject combinations (*i.e.*, subsets of the subjects) were considered exhaustively, and for total number of subjects from one to eight, the combination that results in the highest classification accuracy was identified and reported in [38]. It was observed that using data from multiple subjects may affect the performance by several percents. In fact, as data from more than one subject were made available, some of the classifiers were able to generalize better and their correct classification rates improved.

## 8. Conclusions

In this work, features of gyro signals are extracted with DWT decomposition and given as input to ANNs for leg motion classification. Gyro signals acquired at two different positions of the human leg are employed in classifying the eight different leg motions. To find the most discriminative features of these signals, the raw forms of DWT decomposition coefficients of these signals, their normalized means and variances, energy distribution ratios and their means, variances, minimum, and maximum values, and different combinations of these features are considered as input to ANNs. The ANNs are trained in a supervised fashion using the Levenberg-Marquardt algorithm. Among all the features investigated in this work, the feature set comprised of the normalized variances of DWT decomposition coefficients at the fifth level, consisting of 12 elements, results in the highest classification accuracy and very low network complexity. The EDRs of the DWT decomposition coefficients are also very informative. It would be very time consuming and inefficient to use the raw gyro signals, originally consisting of 1,600 samples per segment, as input to the ANNs for classification, even after downsampling. Training the networks would take too much time and the network complexity would be very high. The selected feature set with only 12 elements considerably reduces the network complexity and achieves an average classification accuracy of 97.6% with the RBO 3.1 wavelet.

The main disadvantage of using wavelet transform pre-processing is that there is not a universal way of choosing the best wavelet type for a particular application. In most cases, the most suitable wavelet can only be found experimentally. In this work, the performances of Haar, Daubechies, Coiflets, Symlets, BO and RBO, and the discrete approximation of Meyer wavelets are compared for leg motion classification. Among these wavelet families, the best classification performance with minimum computational complexity is obtained with the RBO 3.1 wavelet. The effects of random initialization of ANNs and random construction of training and test data sets on classification accuracy are also investigated but the results indicate that these effects are not very significant.

The method proposed here for leg motion classification can be applied in the classification of other types of motions of the human body (e.g., in classifying arm and head motions), or more generally in human activity recognition and rehabilitation. Once the ANNs are trained for different motions or activities, the classification process does not take much time. Therefore, an on-line version of the algorithm can be developed for real-time applications, possibly by implementing some of the processing in the hardware. The design of ANN-based predictors can also be considered as a future research direction.

## Figures and Tables

**Table 1.** Motion classification accuracy with the training and test data, and ANN complexity when the normalized means and variances of DWT decomposition coefficients are used as features.

level	classification accuracy (%)		ANN complexity
	training	test	

1	100	82.4	8:10:8
2	100	83.3	12:8:8
3	100	**87.5**	16:16:8
4	100	86.3	20:8:8
5	100	74.6	24:8:8
6	100	77.7	28:10:8
7	100	75.8	32:10:8
8	100	73.1	36:10:8

**Table 2.** Motion classification accuracy with the training and test data, and ANN complexity when only the normalized variances of DWT decomposition coefficients are used as features.

level	classification accuracy (%)		ANN complexity
	training	test	

1	95.3	78.5	4:10:8
2	99.2	91.3	6:8:8
3	100	93.2	8:8:8
4	100	84.5	10:8:8
5	100	**95.1**	12:8:8
6	100	93.6	14:8:8
7	100	89.8	16:8:8
8	100	84.7	18:8:8

**Table 3.** Motion classification accuracy with the training and test data, and ANN complexity when the normalized variances and the EDRs of the DWT decomposition coefficients are used as features.

level	classification accuracy (%)		ANN complexity
	training	test	

1	98.4	84.8	8:10:8
2	100	89.4	12:10:8
3	100	93.2	16:10:8
4	100	89.4	20:10:8
5	100	**95.1**	24:10:8
6	100	**95.1**	28:10:8
7	100	82.4	32:10:8
8	100	91.2	36:10:8

**Table 4.** Fundamental properties of the basic wavelet families.

wavelet family	filter	symmetry	orthogonality	compact support	fast algorithm

Haar	FIR	symmetric	orthogonal	yes	yes
Daubechies	FIR	asymmetric	orthogonal	yes	yes
Coiflets	FIR	near symmetric	orthogonal	yes	yes
Symlets	FIR	near symmetric	orthogonal	yes	yes
bi-orthogonal	FIR	symmetric	bi-orthogonal	yes	yes
reverse bi-orthogonal	FIR	symmetric	bi-orthogonal	yes	yes
Meyer	IIR	symmetric	orthogonal	no	no
discrete Meyer	FIR	symmetric	orthogonal	yes	yes
Morlet	IIR	symmetric	no	no	no
Mexican hat	IIR	symmetric	no	no	no

**Table 5.** Confusion matrices representing the mean and one standard deviation calculated over 10 iterations when the ANN parameters are randomly initialized and (a) the same training and test data sets are used in each iteration and (b) different training and test data sets are randomly constructed in each iteration.

(a)									
		**classified**							
		M1	M2	M3	M4	M5	M6	M7	M8

**true**	M1	100 *±* 0	0 *±* 0	0 *±* 0	0 *±* 0	0 *±* 0	0 *±* 0	0 *±* 0	0 *±* 0
	M2	0 *±* 0	98.2 *±* 3.8	0 *±* 0	0 *±* 0	0 *±* 0	0 *±* 0	0 *±* 0	1.8 *±* 3.8
	M3	0 *±* 0	0 *±* 0	99.7 *±* 1.0	0.3 *±* 1.0	0 *±* 0	0 *±* 0	0 *±* 0	0 *±* 0
	M4	0 *±* 0	0.6 *±* 1.9	0 *±* 0	95.8 *±* 2.9	3.6 *±* 2.4	0 *±* 0	0 *±* 0	0 *±* 0
	M5	0 *±* 0	0 *±* 0	0 *±* 0	4.8 *±* 2.9	95.2 *±* 2.9	0 *±* 0	0 *±* 0	0 *±* 0
	M6	0 *±* 0	0 *±* 0	0 *±* 0	2.4 *±* 4.0	0.9 *±* 2.0	96.7 *±* 3.9	0 *±* 0	0 *±* 0
	M7	0 *±* 0	0 *±* 0	0 *±* 0	0.9 *±* 1.5	0.3 *±* 1.0	0 *±* 0	98.8 *±* 1.6	0 *±* 0
	M8	0 *±* 0	3.3 *±* 4.2	0 *±* 0	0 *±* 0	0 *±* 0	0 *±* 0	0 *±* 0	96.7 *±* 4.2

(b)									
		**classified**							
		M1	M2	M3	M4	M5	M6	M7	M8

**true**	M1	100 *±* 0	0 *±* 0	0 *±* 0	0 *±* 0	0 *±* 0	0 *±* 0	0 *±* 0	0 *±* 0
	M2	0 *±* 0	97.9 *±* 1.5	0 *±* 0	0.3 *±* 1.0	0 *±* 0	0 *±* 0	0 *±* 0	1.8 *±* 1.6
	M3	0 *±* 0	0 *±* 0	99.7 *±* 1.0	0.3 *±* 1.0	0 *±* 0	0 *±* 0	0 *±* 0	0 *±* 0
	M4	0 *±* 0	0 *±* 0	0 *±* 0	92.0 *±* 6.8	7.7 *±* 6.8	0 *±* 0	0.3 *±* 1.0	0 *±* 0
	M5	0 *±* 0	0 *±* 0	0 *±* 0	4.3 *±* 4.8	95.7 *±* 4.8	0 *±* 0	0 *±* 0	0 *±* 0
	M6	0 *±* 0	0 *±* 0	0 *±* 0	1.2 *±* 2.6	0 *±* 0	98.8 *±* 2.6	0 *±* 0	0 *±* 0
	M7	0 *±* 0	0 *±* 0	0.3 *±* 1.0	1.6 *±* 4.9	1.5 *±* 1.6	0 *±* 0	96.6 *±* 4.5	0 *±* 0
	M8	0 *±* 0	5.2 *±* 6.6	0 *±* 0	0 *±* 0	0 *±* 0	0 *±* 0	0 *±* 0	94.8 *±* 6.6

## References

[1] Akyıldız I.F., Su W., Sankarasubramaniam Y., Çayırcı E. (2002). A survey on sensor networks. IEEE Commun. Mag.

[2] García-Hernández C.F., Ibargüengoytia-González P.H., García-Hernández J., Pérez-Díaz J.A. (2007). Wireless sensor networks and applications: A survey. Int. J. Comput. Sc. Netw. Secur.

[3] Lo B., Thiemjarus S., King R., Yang G., Gellersen H.W., Want R., Schmidt A. Body sensor network—A wireless sensor platform for pervasive healthcare monitoring.

[4] Otto C., Milenkovic A., Sanders C., Jovanov E. (2006). System architecture of a wireless body area sensor network for ubiquitous health monitoring. J. Mobile Multimedia.

[5] Drude S. Requirements and application scenarios for body area networks.

[6] Tunçel O., Altun K., Barshan B. (2009). Classifying human leg motions with uniaxial piezoelectric gyroscopes. Sensors.

[7] Altun K., Barshan B., Tunçel O. (2010). Comparative study on classifying human activities with miniature inertial and magnetic sensors. Pattern Recogn.

[8] Jovanov E., Milenkovic A., Otto C., de Groen P.C. (2005). A wireless body area network of intelligent motion sensors for computer assisted physical rehabilitation. J. NeuroEng. Rehab.

[9] Bouten C.V.C., Koekkoek K.T.M., Verduin M., Kodde R., Janssen J. D. (1997). A triaxial accelerometer and portable data processing unit for the assessment of daily physical activity. IEEE Trans. Biomed. Eng.

[10] Chen K.Y., Bassett D.R. (2005). The technology of accelerometry-based activity monitors: Current and future. Med. Sci. Sport. Exer.

[11] Ylisaukko-oja A., Vildjiounaite E., Mäntyjärvi J. Five-point acceleration sensing wireless body area network—design and practical experiences.

[12] Quwaider M., Biswas S. Body posture identification using hidden Markov model with a wearable sensor network.

[13] Chui C.K. (1992). An Introduction to Wavelets.

[14] Shelley T., Barrett J. (1992). Vibrating gyro to keep cars on route. Eng. Mater. Des.

[15] Murata Manufacturing Co., Ltd (1994). Murata Gyrostar ENV-05A Piezoelectric Vibratory Gyroscope Datasheet.

[16] Mallat S.G. (1999). A Wavelet Tour of Signal Processing.

[17] Mallat S.G. (1989). A theory for multiresolution signal decomposition: The wavelet transform. IEEE Trans. Pattern Anal.

[18] Bai B.C., Farhat N.H. (1992). Learning networks for extrapolation and radar target identification. Neural Netw.

[19] Cohen M., Franco H., Morgan N., Rumelhart D., Abrash V., Hanson S.J., Cowan J.D., Giles C.L. (1993). Context-dependent multiple distribution phonetic modeling with MLPs. Advances in Neural Information Processing Systems.

[20] Narendra K.S., Parthasarathy K. (1991). Gradient methods for the optimization of dynamic systems containing neural networks. IEEE Trans. Neural Networ.

[21] Jordan M.I., Jacobs R.A., Touretzky D.S. (1990). Learning to control an unstable system with forward modeling. Advances in Neural Information Processing Systems 2.

[22] Galicki M., Witte H., Dörschel J., Eiselt M., Griessbach G. (1997). Common optimization of adaptive processing units and a neural network during the learning period: Application in EEG pattern recognition. Neural Netw.

[23] LeCun Y., Boser B., Denker J.S., Henderson D., Howard R.E., Hubbard W., Jackel L.D., Touretzky D.S. (1990). Handwritten digit recognition with a back-propagation network. Advances in Neural Information Processing Systems 2.

[24] Lippman R.P. (1987). An Introduction to Computing with Neural Networks. IEEE Acoust. Speech, Signal Process. Mag.

[25] Haykin S. (1999). Neural Networks: A Comprehensive Foundation.

[26] Bishop C.M. (1995). Neural Networks for Pattern Recognition.

[27] Levenberg K. (1944). A method for the solution of certain non-linear problems in least squares. Q. Appl. Math.

[28] Marquardt D. (1963). An algorithm for least squares estimation on nonlinear parameters. SIAM J. Appl. Math.

[29] Beale M.H., Hagan M.T., Demuth H.B. (2010). MATLAB Neural Networks Toolbox™ 7, User’s Guide.

[30] Duda R.O., Hart P.E., Stork D.G. (2001). Pattern Classification.

[31] Haar A. (1910). Zur theorie der orthogonalen funktionensysteme (On the theory of orthogonal function systems). Math. Ann.

[32] Grossmann A., Morlet J. (1984). Decomposition of Hardy functions into square integrable wavelets of constant shape. SIAM J. Appl. Math.

[33] Meyer Y. (1986). Ondettes, functions splines et analyses graduées, Seminaire EDP.

[34] Mallat S.G. (1987). A compact multiresolution representation: The wavelet model.

[35] Daubechies I. (1988). Orthonormal basis of compactly supported wavelets. Comm. Pure Appl. Math.

[36] Daubechies I. (1992). Ten Lectures on Wavelets.

[37] Grossmann A., Kronland-Martinet R., Morlet J., Combes J.M., Grossmann A., Tchamitchian P. (1989). Reading and understanding continuous wavelet transform. Wavelets: Time-Frequency Methods and Phase-Space.

[38] Altun K., Barshan B., Salah A.A., Gevers T., Sebe N., Vinciarelli A. (2010). Human activity recognition using inertial/magnetic sensor units.

